# Dent disease: clinical practice recommendations

**DOI:** 10.1093/ndt/gfaf003

**Published:** 2025-01-10

**Authors:** Arend Bökenkamp, Gema Ariceta, Detlef Böckenhauer, Olivier Devuyst, Francesco Emma, David van Bennekom, Elena Levtchenko, John Sayer, Aude Servais, Rosa Vargas, Marcin Zaniew, Larisa Prikhodina

**Affiliations:** Department of Pediatric Nephrology, Emma Children's Hospital, Amsterdam University Medical Center, Amsterdam, The Netherlands; Pediatric Nephrology, Hospital Vall d’ Hebron, Autonomous University of Barcelona, Barcelona, Spain; Pediatric Nephrology, University Hospitals Leuven and Department of Cellular and Molecular Medicine, KUL, Leuven, Belgium; Great Ormond Street Hospital for Children and Department of Renal Medicine, UCL, London, UK; Institute of Physiology, University of Zurich, Zurich, Switzerland; UCLouvain Medical School, Brussels, Belgium; Division of Nephrology, Bambino Gesù Children's Hospital, IRCCS, Rome, Italy; European Patient Advocate Group, ERKNet; Department of Pediatric Nephrology, Emma Children's Hospital, Amsterdam University Medical Center, Amsterdam, The Netherlands; Translational and Clinical Research Institute, Newcastle University, Newcastle, UK; Nephrology and Transplantation Department, Inherited Kidney Diseases Reference Center, Necker-Enfants Malades University Hospital, Assistance Publique Hôpitaux de Paris, Inserm U1163, Imagine Institute, Université de Paris, Paris, France; Department of Genetics, European Hospital Georges Pompidou, Paris, France; Department of Pediatrics, University of Zielona Góra, Zielona Góra, Poland; Veltishev Research Clinical Institute for Pediatrics & Pediatric Surgery, Pirogov Russian National Research Medical University, Moscow, Russia

**Keywords:** guidelines, nephrolithiasis, proteinuria, proximal tubule, systematic review

## Abstract

Dent disease is a rare X-linked tubulopathy that is characterized by low-molecular-weight proteinuria associated with hypercalciuria, which may lead to nephrolithiasis, nephrocalcinosis, and kidney failure between the third and fifth decades of life in 30%–80% of affected males. The disease is most often associated with various manifestations of proximal tubular dysfunction. Affected individuals may present nephrotic-range proteinuria which may be misinterpreted and cause diagnostic delay. Due to its rarity, there is limited evidence to guide diagnosis and management. These clinical practice recommendations summarize the current knowledge on Dent disease and provide guidance for diagnosis and management. The recommendations are based on a systematic search of the literature and were endorsed by a Delphi procedure among stakeholders in the field as well as the respective ERA and ESPN working groups.

## INTRODUCTION

Dent disease (DD) is a rare X-linked tubulopathy that is characterized by low-molecular-weight proteinuria associated with hypercalciuria, which may lead to nephrolithiasis, nephrocalcinosis, and kidney failure between the third and the fifth decades of life in 30%–80% of affected males [1]. The disease is most often associated with various manifestations of proximal tubule dysfunction and is frequently complicated by rickets or osteomalacia.

There is genetic heterogeneity for DD, with ∼50%–60% of patients harbouring pathogenic variants in *CLCN5* (chloride channel 5 gene; classified as Dent disease 1, DD1), some 15% with pathogenic variants in *OCRL* (oculo-cerebrorenal syndrome of Lowe gene, classified as Dent disease 2, DD2) and the remaining 25%–35% of patients having neither identifiable *CLCN5* nor *OCRL* variants. *De novo* variants account for ∼12% of DD1 cases [2] and up to 30% of cases with DD2 [3].

### Prevalence

The exact prevalence of DD is unknown. Based on nationwide registries from Japan (91 patients) [4], France (108 patients) [2], and Great Britain (62 patients) [5], a prevalence of between 1 in 400 000 and 1 in 1 000 000 may be estimated. Still, the true prevalence of DD is probably higher due to the variable phenotype and insidious disease course.

### Pathophysiology

DD1 (MIM #300 009) is caused by inactivating variants in the *CLCN5* gene (located on Xp11.22), which encodes the electrogenic 2Cl^−^/H^+^ exchanger ClC-5 [2, 6, 7]. The clinical presentation of DD1 as a proximal tubulopathy reflects the predominant expression of ClC-5 in the early endosomes of the subapical compartment of proximal tubular cells [8]. Studies in *Clcn5* knock-out and knock-in (KI) mice have demonstrated that inactivation of ClC-5 is associated with a severe trafficking defect in proximal tubular cells, with loss or reduced levels of megalin and cubilin at the brush border, impaired endocytosis and lysosomal processing of endocytosed ligands, and defective internalization of various apical transporters [9–12]. The rescue effect of bone marrow transplantation in *Clcn5* knock-out mice substantiated the link between ClC-5 expression, receptor-mediated endocytosis, and proximal tubular dysfunction [13]. As low-molecular-weight proteins are reabsorbed by receptor-mediated endocytosis in proximal tubular cells, low-molecular-weight proteinuria is an obligate finding in DD.

DD2 (MIM #300 555) defines patients with DD with pathogenic variants in the *OCRL* gene, which also causes the oculo-cerebrorenal syndrome of Lowe [14, 15]. Extrarenal manifestations of patients with DD2 are very mild compared with full-blown Lowe syndrome and may include punctuated congenital cataract, mild developmental delay, and short stature [15, 16]. The manifestations of proximal tubular dysfunction overlap in patients with DD1 and DD2 [17].

The fact that variants in *OCRL* generally mimic the proximal tubular dysfunction encountered in DD1 is explained by the association of the OCRL protein with early endosomes, where it acts to maintain low levels of phosphatidylinositol (PI) 4,5-bisphosphate (PI(4,5)P2) for proper endocytic trafficking [18]. *OCRL* encodes the PI(4,5)P2 5-phosphatase OCRL, which controls phosphatidylinositol moieties in the endolysosomal pathway by degrading PI(4,5)P2 [19]. The increase in PI(4,5)P2 levels in early endosomes stimulates uncontrolled actin polymerization into ‘basket’ structures surrounding aberrant organelles, impairing the trafficking of different receptors, including megalin needed for receptor-mediated endocytosis [18, 20], and causing proximal tubular dysfunction [21]. Accumulation of PI(4,5)P2 on autolysosomal membranes is associated with defective autophagic flux and increased levels of autophagosomes, which could be toxic for proximal tubular cells [22].

The mechanisms behind the transition from proximal tubular dysfunction to progressive chronic kidney disease (CKD) in DD remain to be deciphered. Early changes in proximal tubular cells, including proliferation, dedifferentiation, autophagy, and metabolic adaptation, may become maladaptive and promote inflammation and progression of tubulointerstitial fibrosis by various mechanisms [23]. Tubular proteinuria may also play a role, for instance by eliciting stress responses in distal nephron segments [24]. Emerging evidence also suggests that ClC-5 and OCRL may be expressed in human podocytes, potentially explaining the development of focal segmental glomerulosclerosis (FSGS) lesions observed in kidney biopsies of patients with DD1 and DD2 [25, 26].

### Genotype–phenotype correlation

To date, more than 300 distinct disease-causing variants in the *CLCN5* gene have been identified. A link between specific types of variant and different cellular dysfunctions has been shown, but these effects have not been correlated with the phenotypic heterogeneity observed in DD1 patients [27]. There are no clear mutation hotspots as only a small number of recurrent variants has been reported in different geographic areas [2].

Comparing severe *CLCN5* variants (large deletions, frameshift, nonsense, and splice-site) with missense variants, Blanchard *et al.* found no difference in age at diagnosis, estimated glomerular filtration rate (eGFR), proteinuria, or hypercalciuria, indicating that there is no correlation between genotype and phenotype in DD1 [1]. Also, the course of kidney failure may be variable within the same family [27, 28].

However, two recent series suggest some genotype–phenotype correlation in DD1 [29]. Decreased eGFR was more frequent in subjects with *CLCN5* variants affecting the pore or the cystathionine β-synthase (CBS) domain [30]. In another study, 11 out of 13 patients reaching kidney failure had a truncating *CLCN5* variant and two had a missense variant severely impairing chloride–proton exchange [31].

Regarding the *OCRL* gene, a genotype–phenotype correlation is hypothesized because nearly all truncating variants associated with DD2 are located in exons 1–7, which encompass the PH domain, while variants associated with Lowe syndrome are located in exons 8–24 [32]. This may be due to the presence of an additional translation initiation codon in exon 8 [33]. Missense variants associated with DD2 have been found in exons 4–15 [34]. Of note, both a Lowe syndrome and DD2 phenotype have been reported for five *OCRL* variants, arguing against a clear genotype–phenotype effect [32].

## MATERIALS AND METHODS

Development of these clinical practice guidelines was undertaken as an initiative of the ERKNet working group on metabolic disease and the ESPN working group on inherited kidney disease and performed between 2021 and 2023. We followed the RIGHT (Reporting Items for Practice Guidelines in Healthcare) Statement for Practice Guidelines [35]. Two groups were assembled: a core writing group and a voting panel. The core group included paediatric (A.B., D.B., E.L., M.Z., L.P., F.E., G.A., R.V.) and adult (J.S., A.S., O.D.) nephrologists, geneticists (R.V.) and a DD patient representative (D.v.B.). Working groups focusing on specific topics were formed.

PICO questions [patient or population covered, intervention (i.e. treatment or diagnostics), comparator and outcomes] were formulated and addressed in the literature search and formed the basis for the recommendations.

A systematic literature search was performed on 14 January 2022 using the search terms (dent[Title/Abstract]) AND (‘kidney’[All Fields]) OR (‘dent disease’[All Fields]) AND (1992:2022[pdat]), which yielded 363 hits. Each abstract was screened for relevance in answering the PICO questions by two independent reviewers. Papers describing randomized controlled trials, uncontrolled or observational studies, registries, reviews, and case reports were considered if published in English. For background information, laboratory studies were considered as well, as were therapeutic trials in animal models if providing potentially relevant clinical information. A total of 152 publications were selected for full text review.

Recommendations were elaborated and graded by the writing committee following the American Academy of Pediatrics recommendations according to their level of agreement after literature review. Due to the rarity of the disease and the poor level of evidence, many of these statements could not be graded.

The voting group consisted of members with expertise in paediatric and adult DD or genetic testing, including members of the supporting societies and networks. Voting group members were asked by use of an e-questionnaire to provide a level of agreement on a five-point scale (strongly disagree, disagree, unsure, agree, or strongly agree) (Delphi method). A minimum level of 70% agreement was required for final adoption of recommendations.

## DIAGNOSIS

**Figure 1:**
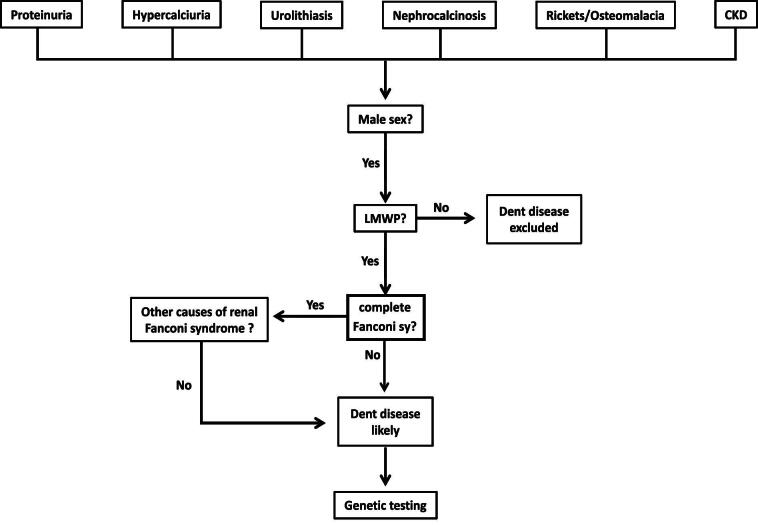
Flow diagram for the diagnosis of Dent disease. LMWP, low-molecular-weight proteinuria.

The diagnosis of DD can easily be missed because of the absence of obvious diagnostic symptoms. Patients may present with incidentally noted proteinuria, which can be very significant, reaching several grams per day, sometimes leading to an erroneous diagnosis of ‘steroid-resistant nephrotic syndrome’ [36]. Other subjects may present with rickets or urolithiasis with or without nephrocalcinosis, or simply with previously undiagnosed CKD [37]. Thus, a high index of suspicion is needed to make the diagnosis. Proteinuria without nephrotic syndrome (normal plasma albumin, no oedema), hypercalciuria/nephrocalcinosis/urolithiasis, hypokalaemia, or unexplained CKD in a male patient are all symptoms that should prompt consideration of the diagnosis, especially in the context of a history of kidney failure in male relatives on the maternal side. Once suspected, the diagnosis is strongly supported by the presence of low-molecular-weight proteinuria and should be confirmed by genetic testing. A diagnostic algorithm is presented in Fig. 1.

All patients with DD have low-molecular-weight proteinuria and screening for this is the first crucial step in establishing the diagnosis (for details, see section Proteinuria, below). The second most frequent urinary finding is hypercalciuria. Although urinary calcium excretion may be normal in some cases, low-normal values are not observed. Age-specific reference values for calcium/creatinine ratio have been published elsewhere [38, 39].

Thus, the combination of low-molecular-weight proteinuria and hypercalciuria strongly suggests a diagnosis of DD. Still, regional differences need to be considered: while hypercalciuria has been reported in more than 90% of patients from Europe and North America, its prevalence is only around 50%–70% in series from Japan, Korea, and China [4, 40, 41].

Generalized proximal tubular dysfunction (i.e. complete renal Fanconi syndrome) is usually not observed. Table 1 lists findings and their frequency as reported in the literature.

**Table 1:** Frequency (%) of selected symptoms of DD1 and DD2 (data from [1, 2, 4, 15, 17, 27, 28, 30, 33, 34, 36, 41–44]).

	**Dent disease 1**	**Dent disease 2**
Kidney abnormality		
Low-molecular-weight proteinuria	100	100
Hypercalciuria	44–90	80–100
Nephrolithiasis	20–40	10–15
Nephrocalcinosis	40–75	10–40
Incomplete Fanconi syndrome	25–65	30–70
Aminoaciduria	20–50	40–70
Hypokalaemia	20–40	10–20
Glucosuria	20–40	5–15
Hypophosphatemia	15–35	10–20
Metabolic acidosis	5–15	5–25
Kidney cysts (cortex and medulla)	33	
Extrarenal abnormality		
Growth retardation	10–20	60–80
Rickets	5–33	10–20
Intellectual impairment	0–9	25–30
Congenital cataract	Very rare	7–10
Elevated serum levels of CPK, ASAT and/or LDH	5–20	80–90

CPK, creatine phosphokinase; ASAT, aspartate aminotransferase; LDH, lactate dehydrogenase.

In the first detailed description of the clinical features of DD by Wrong *et al.*, a urinary concentrating defect was also described [44]. However, reported urinary osmolalities were typically between 300 and 400 mosm/kg and were obtained mostly in adult patients with advanced CKD.

The disease may remain asymptomatic, and patients may be diagnosed only in adulthood. Systematic measurement of low-molecular-weight proteinuria in male adults with unexplained CKD usually allows consideration of the diagnosis. Genetic testing is indicated in all patients for confirmation of the diagnosis.

**Figure 2:**
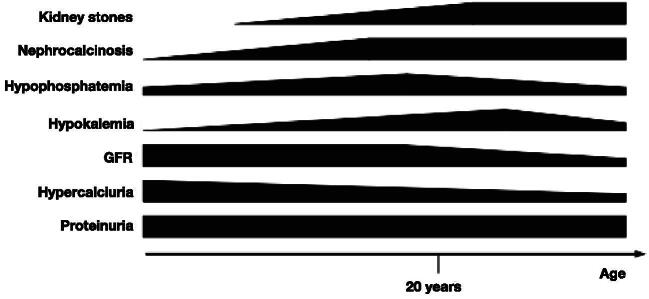
Time course of disease manifestations (schematic presentation illustrating changes in prevalence/severity over time as described in the text).

As presented in Fig. 2, the manifestations of DD are age-dependent to some extent. While proteinuria is present during the entire course of the disease, hypercalciuria is detected in 61%–73% of children and young adults compared with 14%–19% of adults [1, 30]. This decrease is possibly related to the decline in GFR [1]. Nephrocalcinosis is often detectable from childhood while kidney stones manifest at a later age [30].

Low plasma phosphate concentrations are found even in advanced stages of CKD [1]. Plasma potassium concentration decreases with age (0.1 mmol/L per 10 years) and half of patients older than 18 years have hypokalaemia (<3.5 mmol/L) [1]. This decline in plasma potassium concentration is observed despite the decrease in eGFR. Also, plasma bicarbonate remains normal in most subjects until CKD stage 5 [1].

Slowly progressive loss of kidney function with a mean decline of ∼1.5 mL/min/1.73 m^2^ per year is another hallmark of DD [1]. In two large European series median [30]/mean [1] age in patients with normal eGFR was 9.5 years, whereas age of patients with CKD2, CKD3, CKD4, and CKD5 was 13.2/14.1, 17/25.0, 33.0/35.3, and 51.5/39.9 years, respectively. Four out of ten patients aged 30–40 years, three out of nine aged 40–50 years, and three out of four aged 50–60 years had kidney failure [1].

## DIFFERENTIAL DIAGNOSIS

Due to the variability in presentation, the differential diagnosis for DD is extremely broad. We therefore recommend genetic testing as gold standard in each case suspected of DD. Once low-molecular-weight proteinuria has been established, the differential diagnosis mainly includes other causes of proximal tubular dysfunction, both genetic and acquired (Table 2). Variable degrees of proximal tubular dysfunction, including phosphate, bicarbonate, and amino acid wasting, can be observed, but patients rarely present a complete renal Fanconi syndrome. In particular, glucosuria is absent in most cases, or only mild.

**Table 2:** Differential diagnosis of proximal renal tubular dysfunction. list of inherited and acquired aetiologies of proximal renal tubular dysfunction [45–49].

**Disease**	**Characteristic features**
Inherited (gene)	
Nephropathic cystinosis (*CTNS*)	Poor growth, rickets, corneal cystine crystals, CKD
Lowe syndrome (*OCRL*)	Congenital cataracts, glaucoma, general hypotonia, mental retardation, CKD
Galactosaemia (*GALT, GALK1, GALE, GALM*)	Presentation in the neonatal period after ingestion of galactose, jaundice, liver disease, food intolerance (vomiting, diarrhoea), hypoglycaemia, cataract, encephalopathy
Tyrosinaemia (*FAH*)	Poor growth, liver disease
Fructose intolerance (*ALDOB*)	Vomiting, hypoglycaemia, liver disease
Wilson disease (*ATP7B*)	Liver disease, encephalopathy, Kayser–Fleischer rings
Mitochondrial cytopathies (multiple nuclear and DNA mitochondrial variants)	Multisystem dysfunction (brain, muscle, liver, heart, CNS)
Arthrogryposis–renal–cholestasis (ARC) syndrome (*VPS33B, VIPAS39*)	Congenital joint contractures, cholestasis, ichthyosis, platelet abnormalities, dysmorphic features
Fanconi–Bickel syndrome (*GLUT2*)	Failure to thrive, hypoglycaemia, rickets, liver disease
Fanconi renotubular syndrome 1 (*GATM*)	Slowly progressive CKD, autosomal dominant inheritance
Fanconi renotubular syndrome 2 (infantile hypercalcaemia type 2) (*SLC34A1*)	Hypercalcaemia, elevated 1,25(OH)_2_ vitamin D, decreased serum PTH
Fanconi renotubular syndrome 3 (*EHHADH*)	Rickets, short stature
Fanconi renotubular syndrome 4 with maturity-onset diabetes of the young (MODY) (*HNF4A*)	Neonatal hyperinsulinism, MODY, macrosomia
Fanconi renotubular syndrome 5 (*NDUFAF6*)	Rickets, short stature, pulmonary disease, CKD, specific founder variant
Lysinuric protein intolerance (*SLC7A7*)	Protein intolerance (vomiting, diarrhoea), failure to thrive, hyperammonaemia, respiratory insufficiency, immunological disorders
Imerslund–Gräsbeck syndrome (*CUBN; AMN)*	Albuminuria, vitamin B 12-dependent megaloblastic anaemia, failure to thrive, neurological abnormalities May also present without B12 deficiency
Donnai–Barrow syndrome (*LRP2*)	Severe myopia (>6 dioptre), retinal anomalies, hypertelorism, sensorineural deafness, congenital diaphragmatic hernia, agenesis of the corpus callosum, developmental delay
Cholestasis, progressive familial intrahepatic 10 (*MYO5B*)	Cholestasis, jaundice, pruritus, hepatomegaly
Acquired	
Tubulointerstitial nephritis	May be associated with uveitis (TINU syndrome)
Drug-induced (aspirin, didanosine, lamivudine, stavudine, ifosfamide, oxaplatin, cisplatin, valproic acid, aminoglycosides, expired tetracyclines, tenofovir, adefovir, cidofovir, sumarin, fumaric acid, deferasirox, imatinib mesylate, lenalidomide)	
Heavy metal exposure (lead, iron, cadmium, copper, mercury)	Nausea, persistent vomiting, diarrhoea, abdominal pain, encephalopathy, cardiomyopathy, acute tubular necrosis, metabolic acidosis
Sjӧgren's syndrome	Dry eyes, dry mouth, dry skin, muscle or joint pain, salivary gland enlargement, rashes, lymphadenopathy, pulmonary, gastrointestinal, cardiac, neurological, haematological involvement, presence of anti-SSA/SSB and rheumatoid factor
Monoclonal gammopathies, including multiple myeloma	Adults with bone pain, pathological fractures, weakness, anaemia, hypercalcaemia, CKD; clonal bone marrow plasma cells
Paroxysmal nocturnal haemoglobinuria	Renal cortical haemosiderosis, dermatological, gastrointestinal, immunological, and haematological (anaemia, haemorrhage) disorders, AKI due to intravascular haemolysis, CKD

CNS, central nervous system; MODY, maturity onset diabetes of the young; SSA, soluble substance A; SSB, soluble substance B; TINU, tubulointerstitial nephritis and uveitis.

Genetic testing facilitates a definitive diagnosis of DD.

## CLINICAL AND BIOCHEMICAL ASSESSMENT

### Proteinuria

We recommend measurement of low-molecular-weight proteinuria in a male with persisting, non-orthostatic proteinuria (Grade B, strong).

#### Comment

Proteinuria in DD primarily reflects the impaired reabsorption of filtered proteins in the proximal tubule. Proteinuria is thus ‘tubular’ in origin and predominated by low-molecular-weight proteinuria. Nevertheless, proteinuria in the nephrotic range is observed in about one-half of DD patients and includes substantial amounts of albumin, probably reflecting the albumin that is physiologically filtered and not reabsorbed in the proximal tubule [50]. Therefore, plasma albumin levels are typically normal or near normal and patients with DD do not have nephrotic syndrome (see also section Diagnosis, above) [36].

The choice of marker for low-molecular-weight proteinuria depends mainly on local availability. Retinol-binding protein has been reported to have the highest sensitivity and specificity for tubular proteinuria [51]. β_2_-Microglobulin may be degraded by bacteria in the urine and is unstable in acidic pH [52]. Age-specific reference values for albumin/creatinine and low-molecular-weight protein/creatinine should be used [53]. Commonly, at least a 5-fold increase is regarded as diagnostic, although it will be much higher in most patients. An α_1_-microglobulin/creatinine ratio above 120 mg/g (13.6 mg/mmol) has high accuracy in separating DD from glomerular disease [54]. The use of bivariate graphs plotting the urinary low-molecular-weight protein/creatinine ratio against the albumin/creatinine ratio is very helpful to identify patients with DD and female carriers [51].

If specific low-molecular-weight proteinuria markers are not available, a urine protein electrophoresis or the ratio between albumin and total protein in the urine can be used as an indirect indicator for a glomerular or tubular aetiology of the proteinuria: glomerular proteinuria is non-specific and thus the ratio of albumin to total protein in the urine should be roughly the same as in plasma (i.e. >40%). In contrast, in tubular proteinuria albumin typically makes up <40% of total proteins in the urine [55]. An albumin/total protein ratio cut-off of 21% had acceptable accuracy to differentiate DD from glomerular proteinuria [54].

### Hypercalciuria

Hypercalciuria is not mandatory for the diagnosis of DD but in combination with low-molecular-weight proteinuria in a male highly is suggestive of DD, which should be confirmed or excluded by genetic testing (Grade B, strong).

#### Comment

Although hypercalciuria is the second most frequent finding in DD, its absence does not exclude a diagnosis of DD. Hypercalciuria has been more commonly reported in series from Europe and North America and appears to be less prevalent in Asian patients [4, 40, 41]. Also, the presence of hypercalciuria decreases with age in parallel with the deterioration of kidney function [1, 30].

### Kidney biopsy

We recommend not to perform kidney biopsy in patients with DD. Low-molecular-weight proteinuria should be excluded before performing a kidney biopsy in patients with nephrotic-range proteinuria and normal serum albumin. (Grade B, strong).

#### Comment

Kidney biopsy is sometimes performed in patients with proteinuria if the diagnosis of DD has not been considered. Systematic screening for low-molecular-weight proteinuria in male patients with proteinuria but normal or near-normal plasma albumin levels can raise the suspicion of DD and thus eliminate non-informative kidney biopsies.

Exceptionally, a kidney biopsy may be considered if genetic testing is not available to exclude other renal conditions that warrant specific treatments.

When performed, the kidney biopsy usually shows interstitial fibrosis and mostly global rather than focal segmental glomerulosclerosis [36]. The degree of global sclerosis is correlated with age and kidney function at the time of biopsy but has no prognostic value [56]. In ∼20% of biopsies calcinosis is found [36].

### Bone health

We recommend monitoring bone health biochemically (serum calcium, phosphate, alkaline phosphatase, bicarbonate, 25-OH vitamin D, and PTH) and performing hand X-ray in case of abnormal blood tests in children with DD (Grade B, moderate).

#### Comment

Few data on bone health in DD have been reported, except that some patients present with vitamin D-resistant rickets [44, 57, 58]. Multiple factors may contribute to impaired bone health in this disorder, including chronic hypercalciuria, hyperphosphaturia, acidosis, and CKD.

### Dent disease 1 versus Dent disease 2

We recommend genetic testing to confirm a diagnosis of DD in males and include both *CLCN5* and *OCRL* genes to differentiate between DD1 and DD2 (Grade B, strong).

#### Comment

Some findings are significantly more pronounced in DD2 disease (Table 1). Most patients with DD2 disease have activities of plasma lactate dehydrogenase (LDH), aspartate aminotransferase (ASAT) and creatine phosphokinase (CPK) above the normal range (although less than patients with Lowe syndrome [16]). If present, congenital cataract strongly indicates DD2. Mild intellectual impairment and short stature (average height SD = −2.1) also favour the diagnosis of DD2 [17].

Proteinuria is similar in DD1 and DD2. A number of papers suggest that kidney function impairment starts earlier in DD2 than in DD1 [17, 33, 34, 59, 60], but this was not observed by others [1].

### Female carriers

We suggest evaluation of female carriers of DD at least once at adult age (kidney ultrasound, urine calcium/creatinine ratio, low-molecular-weight proteinuria and kidney function) (Grade C, moderate).

#### Comment

More than one-half of female carriers have low levels of low-molecular-weight proteinuria [2, 51]. About 30% have hypercalciuria and some 20% develop nephrocalcinosis and kidney stones later in life [2, 59] and incidentally CKD [61]. Due to biased X-inactivation, female carriers can rarely experience typical symptoms of DD [62].

## GENETIC TESTING

We recommend genetic testing of the *CLCN5* and *OCRL* genes to confirm the clinical diagnosis of DD1 or DD2 in the following situations (Grade B, strong):

1. Males with isolated and persistent low-molecular-weight proteinuria, or mixed proteinuria in the nephrotic range.
2. Males of all ages with persistent low-molecular-weight proteinuria associated with any additional features of proximal tubular dysfunction, nephrolithiasis/nephrocalcinosis, rickets, and/or CKD.
3. Male patients with CKD of unknown origin.

### Comment

The identification of a hemizygous disease-causing variant in *CLCN5* or *OCRL* genes establishes the diagnosis of DD1 or DD2, respectively. Nevertheless, in ∼20%–25% of cases with clinical diagnosis the genetic test is negative, indicating that further genetic heterogeneity of DD is likely to exist [27]. Therefore, a negative genetic test does not rule out the diagnosis of DD. In advanced stages of CKD, only genetic testing may allow making the diagnosis.

The molecular genetic confirmation of the clinical diagnosis benefits the patients and their families as (i) it can be the end of a diagnostic odyssey, (ii) allows setting up adequate follow-up to manage hypertension, kidney stones, CKD, and kidney failure, (iii) allows genetic counselling, and (iv) allows specific follow-up of potential extra-renal manifestations in patients with DD2.

We recommend performing genetic testing in relatives of males with DD as follows:

1. Mothers in order to determine whether the mother is a heterozygous carrier or if the variant is *de novo* (Grade X, strong).
2. Female relatives of childbearing age from the heterozygous female line for identification of carrier status (Grade X, strong).
3. Genetic confirmation can be considered in brothers of the index case when the mother is a heterozygous carrier and in whom low-molecular-weight proteinuria is detected (Grade X, moderate).

All patients should be offered clinical genetic counselling as a basis for shared decision-making.

### Comment

If the pathogenic *CLCN5* or *OCRL* variant in the family is known, molecular genetic testing can be used to clarify the genetic status of at-risk relatives. This information can support decision-making regarding family planning and reproductive options as well as potential living kidney donation (cf. below) [63].

Confirmation of a carrier status allows early screening of all male offspring. If the mother of the affected male carries a heterozygous disease-causing variant, the chance of transmitting it is 50% in each pregnancy.

Detection of a *de novo* variant can allow reassurance of any siblings and help with the interpretation of variants of unknown significance. With genetic testing of the additional relatives, it is possible to identify the family member in whom a *de novo* pathogenic variant arose, allowing risk determination in extended families.

All sons of affected fathers will be unaffected while all daughters will be heterozygous carriers. This should be communicated to the families. Pre-symptomatic screening in daughters of heterozygous carries should be delayed until the age of 18 years. It should be borne in mind that the absence of low-molecular-weight proteinuria does not exclude DD carriership.

If the decision is taken not to perform diagnostic screening in childhood, parents should be made aware of their responsibility to inform their children of disease risk when they reach the legal age of majority.

If no variant in *CLCN5* and *OCRL* genes is found, massive parallel sequencing of a panel of genes or a virtual panel applied *in silico* following whole-exome or whole-genome sequencing can be considered [64]. Depending on the technique and the bioinformatics tools used, these panels can also detect copy number variants. A molecular genetics diagnostic panel approach allows the analysis of other genes responsible for renal Fanconi syndrome, nephrolithiasis, nephrocalcinosis, or CKD.

Box 1. Recommendations on diagnosis

**Table:** 

• We recommend measurement of low-molecular-weight proteins in a male with persisting, non-orthostatic proteinuria.	B, strong
• Hypercalciuria is not mandatory for the diagnosis of DD but in combination with low-molecular-weight proteinuria in a male is highly suggestive of DD.	B, strong
• We recommend not to perform kidney biopsy in patients with DD. Low-molecular-weight proteinuria should be excluded before performing a kidney biopsy in patients with nephrotic-range proteinuria and normal serum albumin.	B, strong
• We recommend genetic testing to confirm a diagnosis of DD in males and include both *CLCN5* and *OCRL* genes to differentiate between DD1 and DD2.	B, strong
• We recommend including *CLCN5* and *OCRL* in gene panels of unexplained CKD in males irrespective of age.	B, strong
• We recommend genetic testing in the mother of an index case with DD and in female relatives in the childbearing age to document carrier status.	X, strong
• Genetic confirmation can be considered in brothers of the index case when the mother is a heterozygous carrier and in whom low-molecular-weight proteinuria is detected.	X, moderate
• We recommend monitoring bone health biochemically (serum calcium, phosphate, alkaline phosphatase, bicarbonate, 25OH vitamin D, and PTH) and performing X-hand in case of abnormal blood tests in children.	B, moderate

## PRENATAL DIAGNOSIS

Prenatal testing and preimplantation genetic testing are possible if a disease-causing pathogenic variant in the *CLCN5* or *OCRL* genes has been identified in the family. Female carriers can benefit from these tests depending on local legislation. The final decision is personal after discussion with an expert multidisciplinary team.

## TREATMENT

Mineral losses are typically treated with oral supplements, as in other forms of renal Fanconi syndrome aiming to improve serum concentrations. It is often impossible to normalize serum concentrations.

While progress has been made in the last decades to get more insight into the pathophysiology and natural course of DD, little is known about how to reduce stone formation and how to prevent the progressive loss of kidney function. The insidious decline of GFR over decades has hampered the design of controlled trials in humans addressing hard endpoints. Therefore, treatment recommendations are based on pathophysiological considerations, animal studies in knock-out models, or observations using surrogate endpoints.

### Treatment options that may be considered on a personalized basis

#### Citrate

We suggest that citrate treatment may be considered in patients with DD, in particular in the presence of nephrocalcinosis/stone disease (Grade C, weak).

##### Comment

While the excretion of citrate is normal in the majority of patients [44, 65], citrate excretion in DD is lower compared with other forms of renal Fanconi syndrome [44]. Potassium citrate reduces recurrent calcium oxalate nephrolithiasis in patients with idiopathic hypercalciuria and hypocitraturia [66, 67]. A high-citrate diet has been shown to slow progression of CKD in *Clcn5* knock-out mice with decreasing tubular atrophy and dilatation, interstitial fibrosis, and nephrocalcinosis [68]. There have been no human trials studying the efficacy of citrate supplementation in DD. Nonetheless, potassium citrate is prescribed to 13%–25% of DD patients to prevent nephrolithiasis, improve metabolic acidosis, and potentially slow CKD progression [1, 28, 30, 69].

Despite the fact that the efficacy of citrate is uncertain, we suggest including citrate in the therapeutic armamentarium of DD. As citrate is metabolized to bicarbonate, it can also help to increase serum bicarbonate levels in those patients with acidosis. In patients treated with potassium citrate urinary pH should be monitored, as alkaline urine carries the risk of calcium phosphate precipitation [70, 71]. Other potential side effects include upper gastrointestinal disturbance (stomach pains, bloating, nausea) and rash [66]. Citrate may aggravate the alkalosis in patients with significant hyperaldosteronism.

#### Phosphate supplementation

We suggest treating patients with DD using phosphate salts in case of hypophosphatemia and signs of rickets of osteomalacia (Grade X, weak).

##### Comment

Prolonged hypophosphataemia may cause bone demineralization manifesting as rickets in children or osteomalacia in adults. However, there is no clear correlation between the degree of hypophosphataemia and the presence of rickets in DD [30, 65].

There are no studies addressing phosphate supplementation in DD. As hypophosphataemia in DD is usually mild or moderate, it can be corrected with increased dietary phosphate and/or oral supplementation. It seems reasonable to start with a small amount (e.g. 20 mg/kg/day divided into three or four doses), based on elemental phosphorus. Dosage is titrated based on serum alkaline phosphatase and improvement of rickets and bone deformities. Because of the tubular leak, phosphate supplementation increases urinary phosphate excretion, further increasing the risk of nephrocalcinosis/-lithiasis. In contrast to hypophosphataemic rickets, active vitamin D should not be given because of inherently elevated 1.25(OH)_2_ vitamin D and potential worsening of hypercalciuria. Both hypercalciuria and kidney ultrasound should be monitored during treatment with phosphate supplements.

#### Vitamin D supplementation

We suggest measurement of serum vitamin D level in patients with DD and supplementation if low. Serum 1.25(OH)_2_ vitamin D (if measurement is available), calcium, and hypercalciuria need close monitoring if vitamin D supplements are given (Grade X, weak).

##### Comment

The loss of vitamin D binding protein leads to reduced tubular absorption of 25OH vitamin D, while synthesis of 1.25(OH)_2_ vitamin D is increased [72]. This leads to mildly decreased 25OH vitamin D concentrations, while calcitriol levels remain in the normal–high range even in the late stage of CKD [1, 65]. However, usual vitamin D assays measure total blood concentrations, whereas only the free (unbound) form is biologically active. Because of the decreased levels of vitamin D binding protein, blood levels are difficult to interpret in DD.

No studies in DD have addressed vitamin D supplementation and it is still unclear what level of 25OH vitamin D should be aimed at. As bone health is of great concern in DD it seems reasonable to monitor 25OH vitamin D level, and initiate supplementation with native vitamin D if low according to guidelines for a healthy population. Serum and urine calcium, 25OH vitamin D levels and alkaline phosphatase should be monitored [27]. Active vitamin D supplementation is not indicated as calcitriol levels are high.

#### Vitamin A

We recommend vigilance for ocular symptoms of vitamin A deficiency, which should prompt measurement of retinol levels and supplementation if low (Grade B, moderate).

##### Comment

Retinol-binding protein is lost as part of low-molecular-weight proteinuria. This can lead to vitamin A deficiency [73]. Symptoms of vitamin A deficiency are night-blindness and dry eyes, which should prompt measurement of vitamin A levels if available [74]. Symptoms are reversible with vitamin A repletion [75].

#### Growth hormone therapy

We suggest that treatment with growth hormone (GH) for short stature in patients with DD should only be considered if growth failure persists despite adequate metabolic control or in CKD 3 or higher (Grade D, weak).

##### Comment

Growth is compromised in 27% and 54% of patients with DD1 and DD2, respectively [27]. This may be due to a combination of metabolic abnormalities (i.e. acidosis, hypercalciuria, hypophosphataemia, vitamin D deficiency), and CKD and may also be a mild manifestation of Lowe syndrome in DD2 [17]. Thus, growth velocity needs to be observed under optimal metabolic control before GH treatment might be considered. Standard indications for GH like GH deficiency or advanced CKD apply following existing clinical practice guidelines. There are a few reports indicating improved growth velocity in DD patients [76–79]. However, the short observation period and a lack of final height data limit strong conclusions.

### Treatment options that are generally not indicated

#### Thiazides

We recommend that thiazide treatment should not be used systematically in DD. If prescribed, electrolytes and kidney function need to be monitored closely (Grade B, moderate).

##### Comment

Hypercalciuria is one of the hallmarks of DD and regarded as a major risk factor for nephrocalcinosis and stone formation. The kidney stones are composed of calcium phosphate, calcium oxalate, or a combination of both [44, 65, 80]. Still, nephrolithiasis/nephrocalcinosis has also been observed in the absence of hypercalciuria [40], which might be explained by impaired clearance of microcrystals from the surface of collecting duct cells, as demonstrated *in vitro* [81].

In analogy to idiopathic hypercalciuria, chlorthalidone (25 mg/day) [67] and hydrochlorothiazide (>0.4 mg/kg/day) [68] both alone or in combination with amiloride decreased 24-hour urinary calcium and citrate excretion in DD in short-term studies, while amiloride alone had no effect on hypercalciuria. In retrospective clinical cohorts, thiazides were used in 11%–34% of patients with DD [1, 30]. Still, there are no data on the long-term effect of thiazides on kidney function or stone formation in DD.

A recent randomized controlled trial showed no benefit of hydrochlorothiazide even in high doses of 50 mg/day on recurrence of calcium-containing stones in a non-DD population [82], while treatment was associated with hypovolaemia and electrolyte disturbances, which were also observed in DD patients [83]. DD2 patients are even more susceptible to severe dehydration and acute kidney injury when treated with thiazides [34].

#### ACE inhibitors and angiotensin receptor blockers

We suggest that ACE inhibitors (ACEis) or angiotensin receptor blockers (ARBs) should not be used routinely as nephroprotective treatment in patients with DD (Grade C, moderate).

##### Comment

In recent series, ACEi or ARB use was reported in 11%–42% of DD patients [1, 28, 30, 34, 60]. Nephrotic-range proteinuria, presence of glomerulosclerosis, podocyte effacement [56], and lately the expression of CLCN5 and OCRL in podocytes [25, 26], are arguments brought forward by proponents of this treatment.

However, no studies have addressed the effects of ACEis/ARBs on CKD progression in patients with DD or in animal models. Published case series failed to demonstrate a decrease in proteinuria during ACEi/ARB treatment [1, 36, 60], which is not unexpected considering the tubular (rather than glomerular) origin of proteinuria in DD.

The risk/benefit ratio must be well balanced when treating DD patients with an ACEi/ARB as potential side effects such as hypotension have been reported [60] and there is an increased risk in hypovolaemia [84].

## TRANSPLANTATION

We suggest a very prudent approach when considering kidney donation from female carriers of DD. Donation may be considered in an older female if she is asymptomatic (i.e. has normal eGFR, and no stones/nephrocalcinosis or proteinuria) (Grade X, weak).

### Comment

The disease does not recur after kidney transplantation. Cadaveric donation and living-related donation from a father and an unaffected brother are safe. Unless DD occurred *de novo*, Mothers and 50% of sisters are carriers of the mutated gene. Some show mild proteinuria and hypercalciuria. There is a reasonable concern in considering these subjects as potential donors. Very limited experience exists in using a mother's kidney for living kidney donation, which has been reported in an elderly woman without proteinuria or other signs of DD [85]. When risk is difficult to assess, priority should be given to unrelated kidney transplantation [86].

Box 2. Recommendations on treatment

**Table:** 

• We suggest that citrate treatment may be considered in patients with DD, in particular in the presence of nephrocalcinosis/stone disease.	C, weak
• We suggest treating patients with DD using phosphate salts in case of hypophosphataemia and signs of rickets of osteomalacia.	X, weak
• We suggest measurement of serum vitamin D level in patients with DD and supplementation if low. Serum 1.25(OH)_2_ vitamin D, calcium, and hypercalciuria need close monitoring if vitamin D supplements are given.	X, weak
• We recommend vigilance for ocular symptoms of vitamin A deficiency, which should prompt measurement of retinol levels and supplementation if low.	B, moderate
• We suggest that treatment with growth hormone for short stature in patients with DD should only be considered if growth failure persists despite adequate metabolic control or in CKD 3 or higher.	D, weak
• We recommend that thiazide treatment should not be used systematically in DD. If prescribed, electrolytes and kidney function need to be monitored closely.	B, moderate
• We suggest that ACE inhibitors or angiotensin receptor blockers should not be used routinely as nephroprotective treatment in patients with DD.	C, moderate
• We suggest a very prudent approach when considering kidney donation from female carriers of DD. Donation may be considered in an older female if she is asymptomatic (i.e. has normal eGFR, and no stones/nephrocalcinosis or proteinuria).	X, weak

## FACTORS AFFECTING PROGNOSIS

There is no significant relationship between proteinuria and GFR either in patients with DD1 or with DD2; GFR is comparable between patients with and without nephrotic proteinuria [1, 31, 36]. While glomerular and tubulointerstitial fibrosis are strongly correlated [36, 56], this does not apply to the amount of proteinuria or the rate of decline of kidney function. Mild focal podocyte effacement was correlated with progression of CKD during follow-up [56].

There is no association between nephrocalcinosis and the rate of GFR decline [1, 34, 56]. In patients with DD and medullary nephrocalcinosis, progression to kidney failure was not related to its severity, and in a few patients kidney failure even occurred in its absence [44, 65].

## FOLLOW-UP

We recommend that a tertiary care centre with experience in the diagnosis and treatment of DD should be involved in the care of patients with DD (Grade B, strong).

### Comment

Management of DD patients in shared care between a local nephrologist and a tertiary nephrological unit with experience in DD combines ease of access to adequate healthcare with implementation of new insights in diagnosis and treatment of DD. This will facilitate clinical and genetic diagnosis, patient education, and access to specialist stone urology as well as referral to a paediatric endocrinologist in case of growth failure.

The intensity of clinical and biochemical monitoring depends on the severity of clinical and biochemical abnormalities and is usually at intervals of 3–6 months, while ultrasound is repeated on a yearly basis in the absence of complaints. Table 3 provides guidance on clinical and biochemical parameters and imaging for diagnosis and follow-up.

**Table 3:** Suggested clinical, biochemical and imaging tests for diagnosis and follow-up of DD.

	Initial evaluation	Follow-up
Clinical		
Stones	+	+
Rickets	+	+
Growth failure	+	+
Absence of oedema	+	
Biochemical		
Blood	Na, K, Cl, Ca, PO_4_, HCO_3_, creatinine, urea, albumin, alkaline phosphatase, PTH, 25OH vitamin D	Na, K, Cl, Ca, PO_4_, HCO_3_, creatinine, urea, alkaline phosphatase, PTH, 25OH vitamin D
Urine		
Spot urine	Total protein, low-molecular-weight proteinuria, albumin, calcium, creatinine	Calcium, creatinine
Imaging		
Kidney ultrasound scan	Nephrocalcinosis, nephrolithiasis, cysts	Nephrocalcinosis, nephrolithiasis, cysts
Hand X-ray	Clinical rickets or elevated alkaline phosphatase	Clinical rickets or elevated alkaline phosphatase
Molecular genetics		
*CLCN5, OCRL genes*	+	
Consultations		
Clinical genetics	+	
Ophthalmology	Cataract (if OCRL+)	If ocular symptoms suggestive of vitamin A deficiency

Follow-up may need to be intensified when kidney function declines, as in all other forms of progressive CKD.

Box 3. Recommendations on follow-up

**Table:** 

• We recommend performing kidney ultrasound at diagnosis and at regular intervals during follow-up.	B, strong
• We recommend that a tertiary care centre with experience in the diagnosis and treatment of DD should be involved in the care of patients with DD.	B, strong
• We suggest evaluation of female carriers of DD at least once at adult age (kidney ultrasound, urine calcium/creatinine ratio, low-molecular-weight proteinuria, and kidney function).	C, moderate

## SUGGESTIONS FOR FUTURE STUDIES

While the pathophysiology of DD is understood quite well, there is an urgent need for treatment validation and optimalization. Using data from existing DD registries the short-term efficacy and side effects of anti-proteinuric treatment with ACEis or ARBs might be evaluated. Both treatment modalities could also be evaluated prospectively in animal models in analogy to the study on citrate treatment in knock-out mice [68]. Designing prospective randomized therapeutic trials in humans will be difficult, however, due to the insidious and variable deterioration of kidney function and the absence of a reliable surrogate marker for long-term prognosis.

## CONCLUSIONS

The clinical practice recommendations (Boxes 1–3) reflect the expert consensus opinion of the authors, which were commented on by a large Delphi panel and were endorsed by at least 70% of participants as well as the respective ERA and ESPN working groups.

We acknowledge that there is little evidence to guide treatment of DD and that our recommendations in this respect are based mainly on pathophysiological considerations rather than data from clinical trials. The slow, insidious course of DD is a major obstacle for the design of such trials. Prospective data collection in DD registries in combination with genetic testing is ongoing and may help identify high-risk patients for potential trials in the future [29].

## Data Availability

The data underlying this article will be shared upon reasonable request to the corresponding author.
